# Supplementary material for: Native American ataxia medicines rescue ataxia-linked mutant potassium channel activity via binding to the voltage sensing domain
Source: Nat Commun. 2023 Jun 6;14:3281. doi: 10.1038/s41467-023-38834-6 (PMC10244465; doi:10.1038/s41467-023-38834-6)
Supplement: Supplementary file 1 — Supplementary Information [file 41467_2023_38834_MOESM1_ESM.pdf]

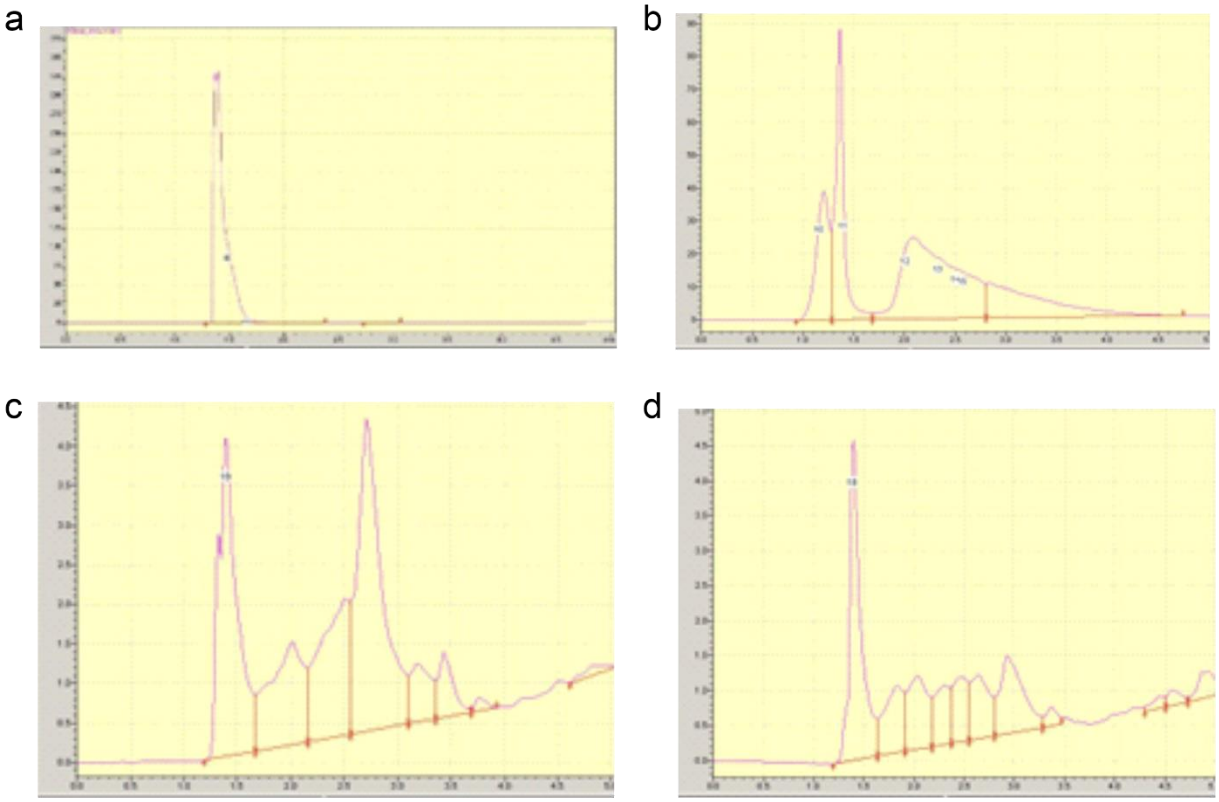

Supplementary Figure 1. Detection of gallic acid in *F. gardneri* and *P. capitatus* extracts.

Analysis of plant extracts after dilution 2- to 10-fold with MeOH was used to detect gallic acid by MS. In negative ionization mode authentic gallic acid showed molecular ion m/z 169 ($M\text{-}H^+$) and daughter ion m/z 125. *F. gardneri* and *P. capitatus (leaves and roots)* gave solutions after dilution in MeOH and were directly injected onto the LC/MS. Gallic acid was identified in extract of *F. gardneri* and *P. capitatus* root.

Preparative RPHPLC of plant extracts (panels A-D). Aqueous plant extracts were diluted with 0.1% TFA/MeOH before injection. If 1:1 dilution didn't give solutions, additional 0.1% TFA/MeOH was added until a clear solution formed.

a. Injection of authentic gallic acid (2.1 mg) in 0.1% TFA/MeOH with mobile phase 15 to 100% MeOH/water (both with 0.1% TFA) gave a single peak with retention time (RT) 1.4 min with UV maximum at 270 nm.
b. Injection of *F. gardneri* extract (0.25 mL diluted with 0.75 mL 0.1% TFA/MeOH) showed peaks at 1.2 min, 1.35 min and a broad peak at RT 2.1 min. Analysis of the peak with RT 1.35 min after 10-fold dilution with MeOH showed the presence of gallic acid.
c. The leaf extract of *P. capitatus* gave peaks with RT 1.2 and 1.4 min but LC/MS analysis did not show gallic acid.
d. Injection of the *P. capitatus* root extract (0.5 mL diluted 1:1 with 0.1% TFA/MeOH) gave a major peak with RT 1.4 min that showed gallic acid by LC/MS after 10-fold dilution with MeOH.

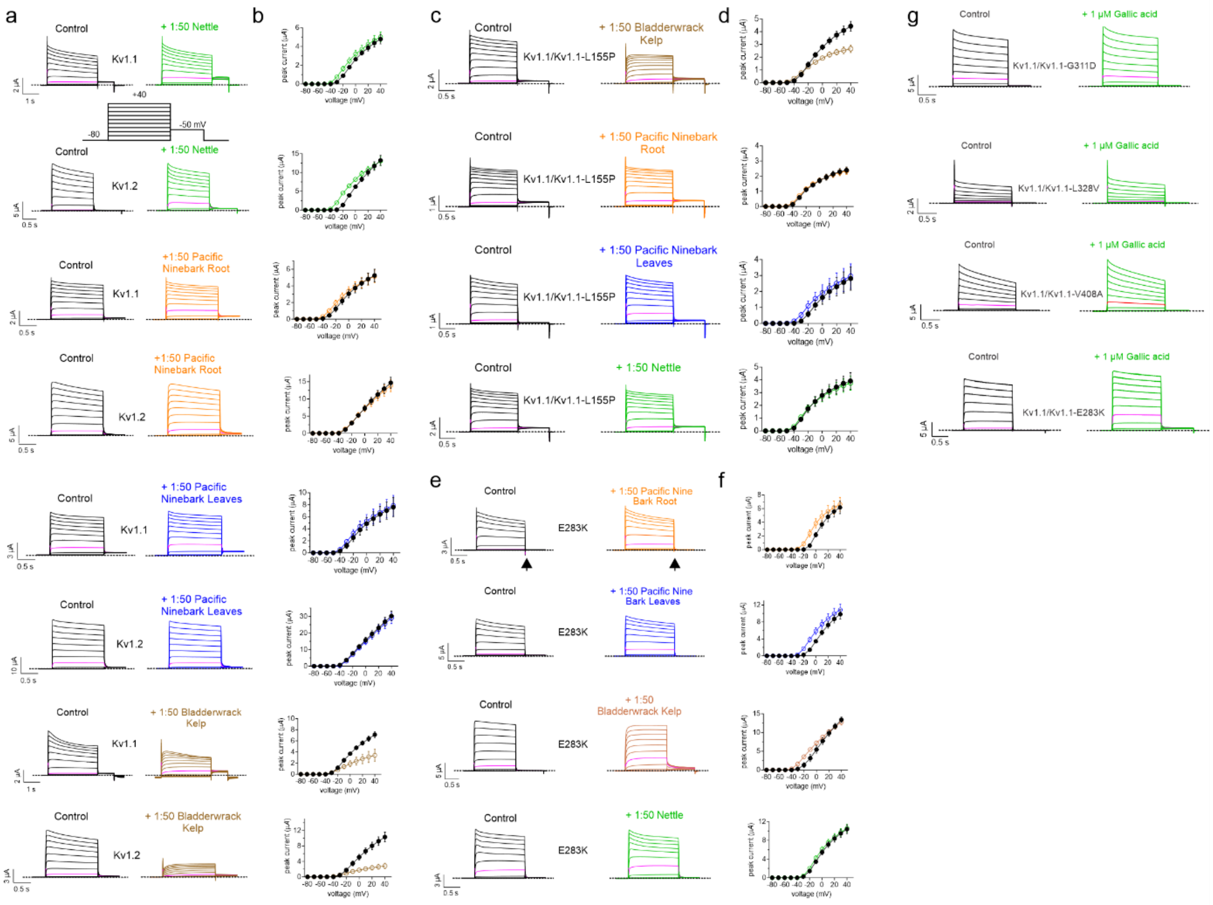

Supplementary Figure 2. Full voltage families and IV plots to supplement main figures.
Voltage protocol as in Figure 2. Error bars indicate SEM. At least 2 batches of oocytes were used per experiment. Magenta traces indicate same-voltage traces within each pairing for ease of visual comparison.

a, b. Full voltage families (a) and IV plots (b) for groups as in Figure 1b-d; Kv1.1 nettles (*n* = 5); Kv1.2 nettles (*n* = 6); Kv1.1 pacific ninebark root (*n* = 5); Kv1.2 pacific ninebark root (*n* = 5); Kv1.1 pacific ninebark leaves (*n* = 5); Kv1.2 pacific ninebark leaves (*n* = 5); Kv1.1 bladderwrack kelp (*n* = 4); Kv1.2 bladderwrack kelp (*n* = 6). Error bars indicate SEM.

c, d. Full voltage families (c) and IV plots (d) for groups as in Figure 5b-d, *n* = 5. Error bars indicate SEM.

e, f. Full voltage families (e) and IV plots (f) for groups as in Figure 6a-c, *n* = 5. Error bars indicate SEM.

g. Full voltage families for groups as in Figure 7a-d, *n* = 5.

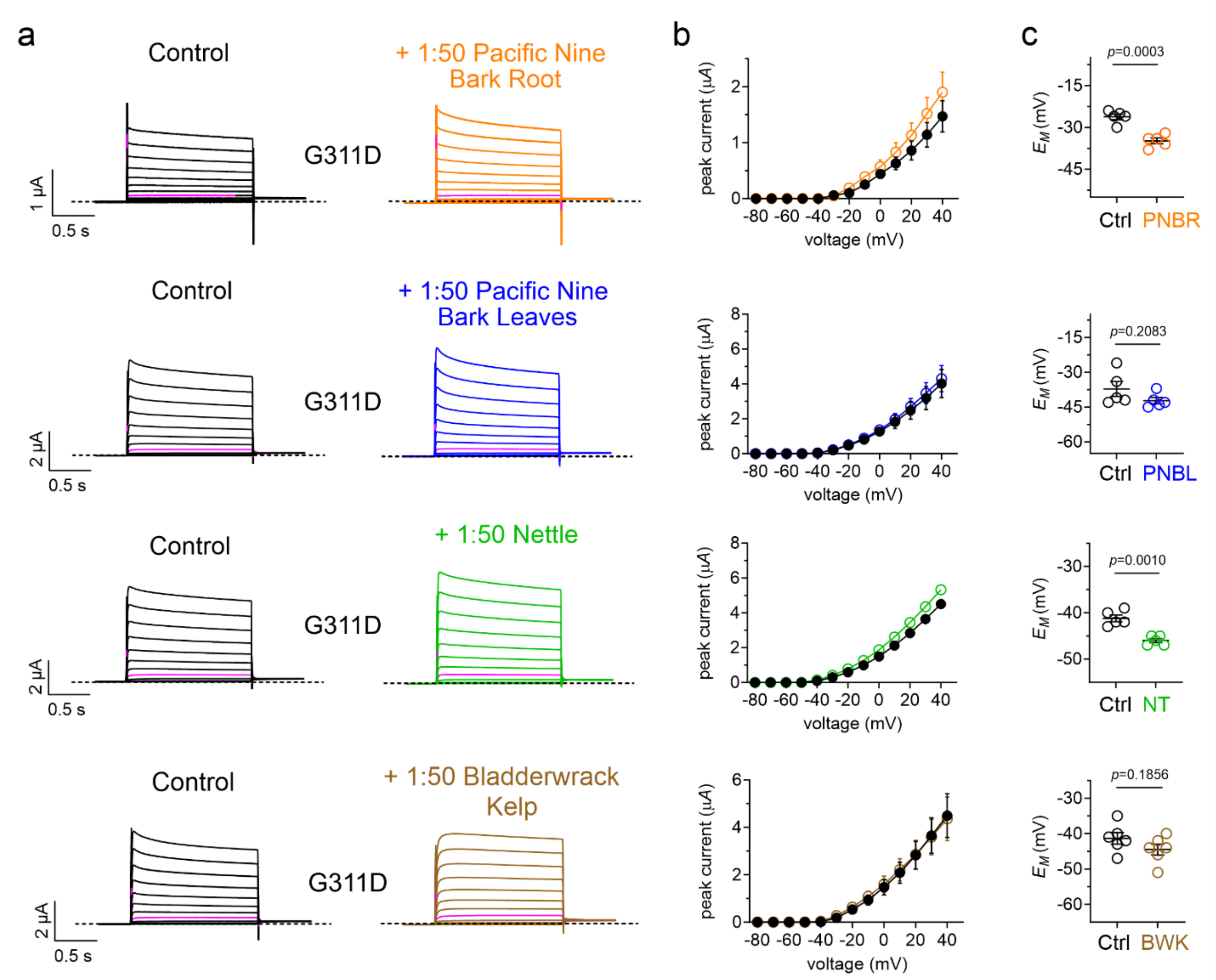

Supplementary Figure 3. Ataxia therapy extracts do not rescue the function of Kv1.1-G311D.
Voltage protocol as in Figure 2. Error bars indicate SEM; statistical analysis by two-tailed paired t-test. At least 2 batches of oocytes were used per experiment. Magenta traces indicate same-voltage traces within each pairing for ease of visual comparison.
a. Mean trace for Kv1.1-G311D in the absence (Control) and presence of plant extracts as indicated (1:50 dilution); n = 5.
b. Mean peak current versus voltage for Kv1.1-G311D traces as in a; n = 5.
c. Mean E_M for oocytes expressing Kv1.1-G311D in the absence (Control) and presence of plant extracts as in A; pacific ninebark root (n = 5; p=0.0003); pacific ninebark leaves (n = 5; p=0.2083); nettle (n = 5; p=0.0010); bladderwrack kelp (n = 5; p=0.1856).

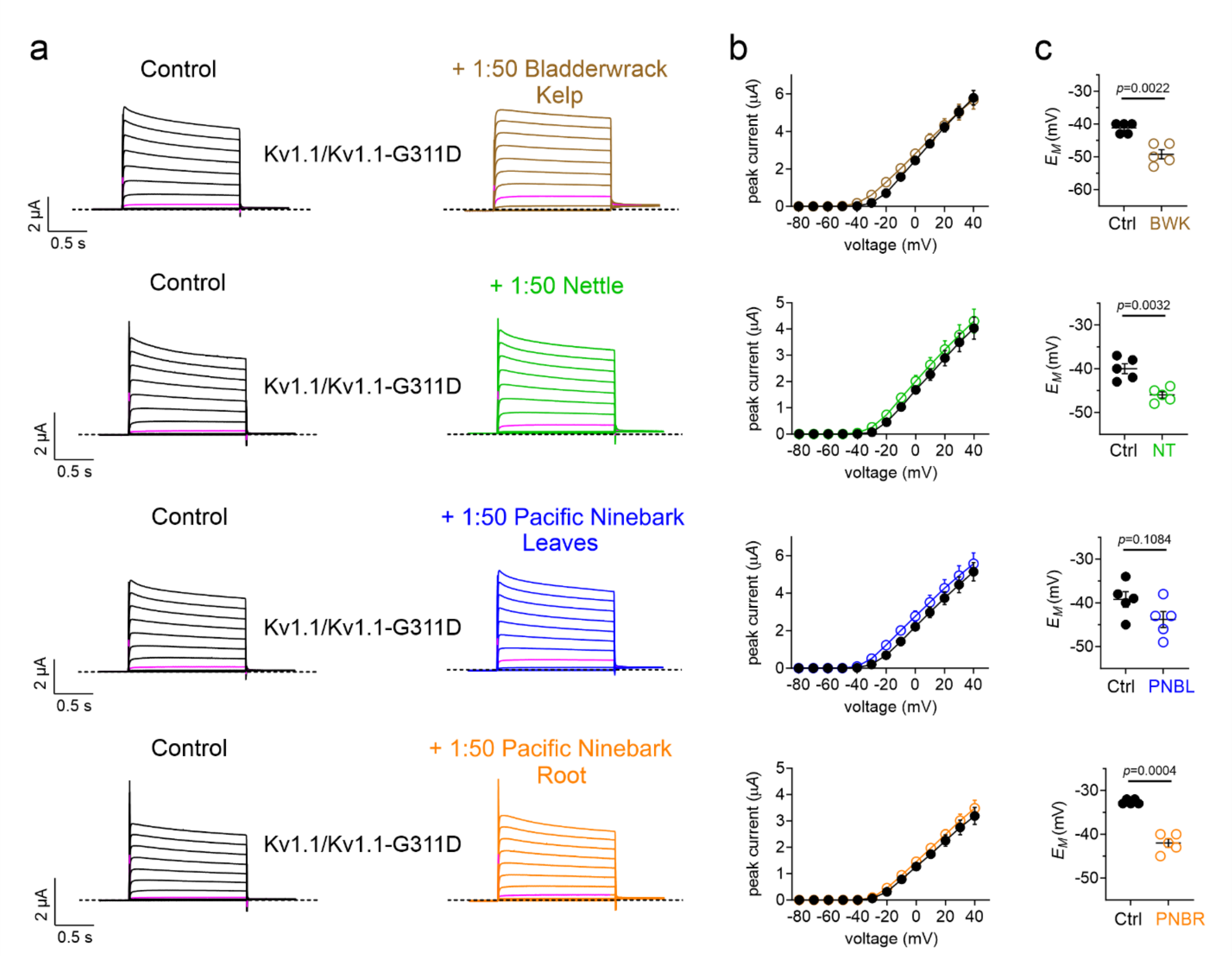

Supplementary Figure 4. Ataxia therapy extracts do not rescue the function of Kv1.1/Kv1.1-G311D. Voltage protocol as in Figure 2. Error bars indicate SEM; statistical analysis by two-tailed paired t-test. At least 2 batches of oocytes were used per experiment. Magenta traces indicate same-voltage traces within each pairing for ease of visual comparison.

a. Mean trace for Kv1.1/Kv1.1-G311D in the absence (Control) and presence of plant extracts as indicated (1:50 dilution); *n* = 5.

b. Mean peak current versus voltage for Kv1.1/Kv1.1-G311D traces as in a; *n* = 5.

c. Mean E_M for oocytes expressing Kv1.1/Kv1.1-G311D in the absence (Control) and presence of plant extracts as in A; bladderwrack kelp (*n* = 5; *p*=0.0022); nettle (*n* = 5; *p*=0.0032); pacific ninebark leaves (*n* = 5; *p*=0.1084); pacific ninebark root (*n* = 5; *p*=0.0004).

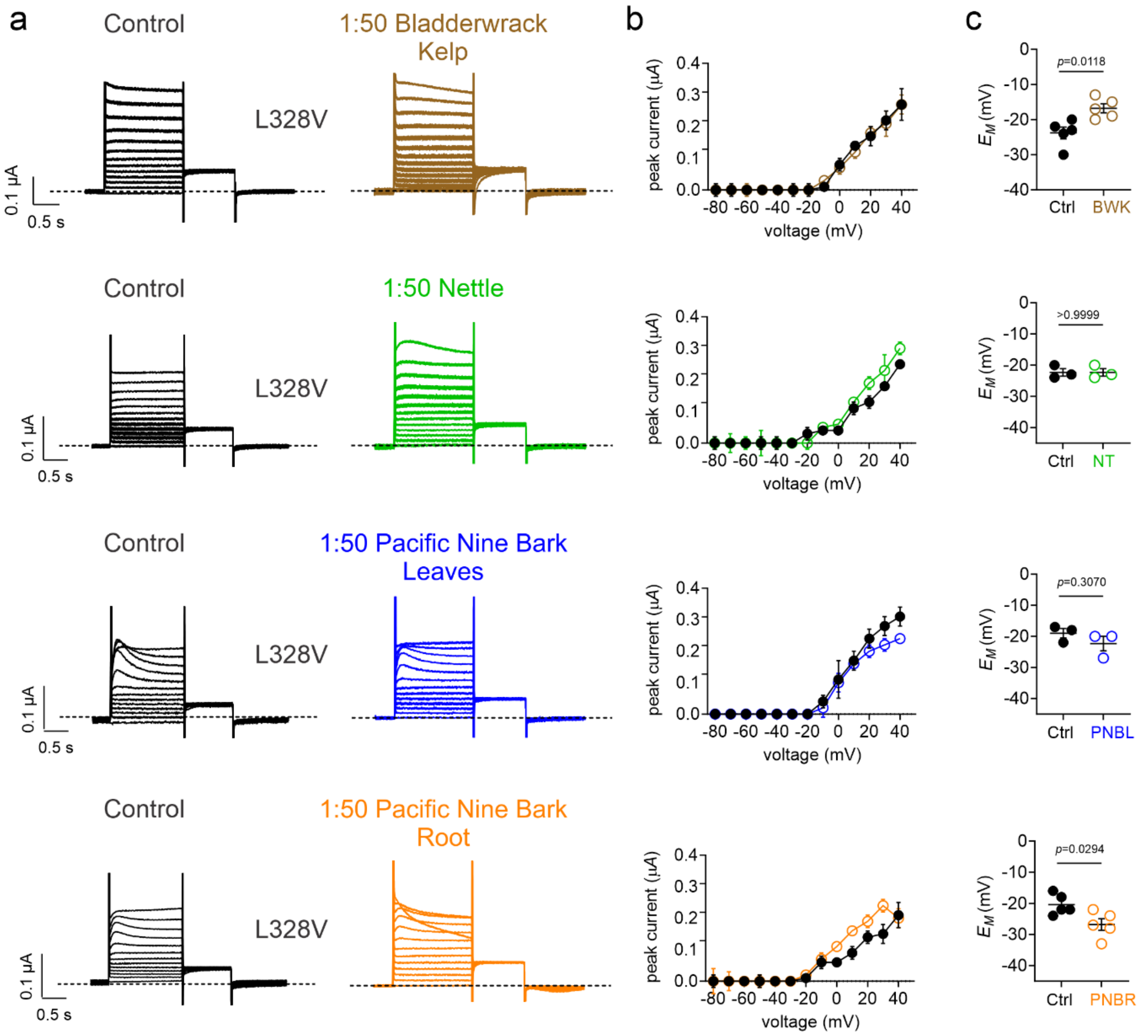

Supplementary Figure 5. Ataxia therapy plant extracts do not rescue the function of Kv1.1-L328V. Voltage protocol as in Figure 2. Error bars indicate SEM; statistical analysis by two-tailed paired t-test. At least 2 batches of oocytes were used per experiment. Magenta traces indicate same-voltage traces within each pairing for ease of visual comparison.

a. Mean trace for Kv1.1-L328V in the absence (Control) and presence of plant extracts as indicated (1:50 dilution). bladderwrack kelp (*n* = 5); nettle (*n* = 3); pacific ninebark leaves (*n* = 3); pacific ninebark root (*n* = 5).

b. Mean peak current versus voltage for Kv1.1-L328V traces in a; bladderwrack kelp (*n* = 5); nettle (*n* = 3); pacific ninebark leaves (*n* = 3); pacific ninebark root (*n* = 5).

c. Mean E_M for oocytes expressing Kv1.1-L328V in the absence (Control) or presence of plant extracts as in a; bladderwrack kelp (*n* = 5; *p*=0.0118); nettle (*n* = 3; >0.9999); pacific ninebark leaves (*n* = 3; *p*=0.3070); pacific ninebark root (*n* = 5; *p*=0.0294).

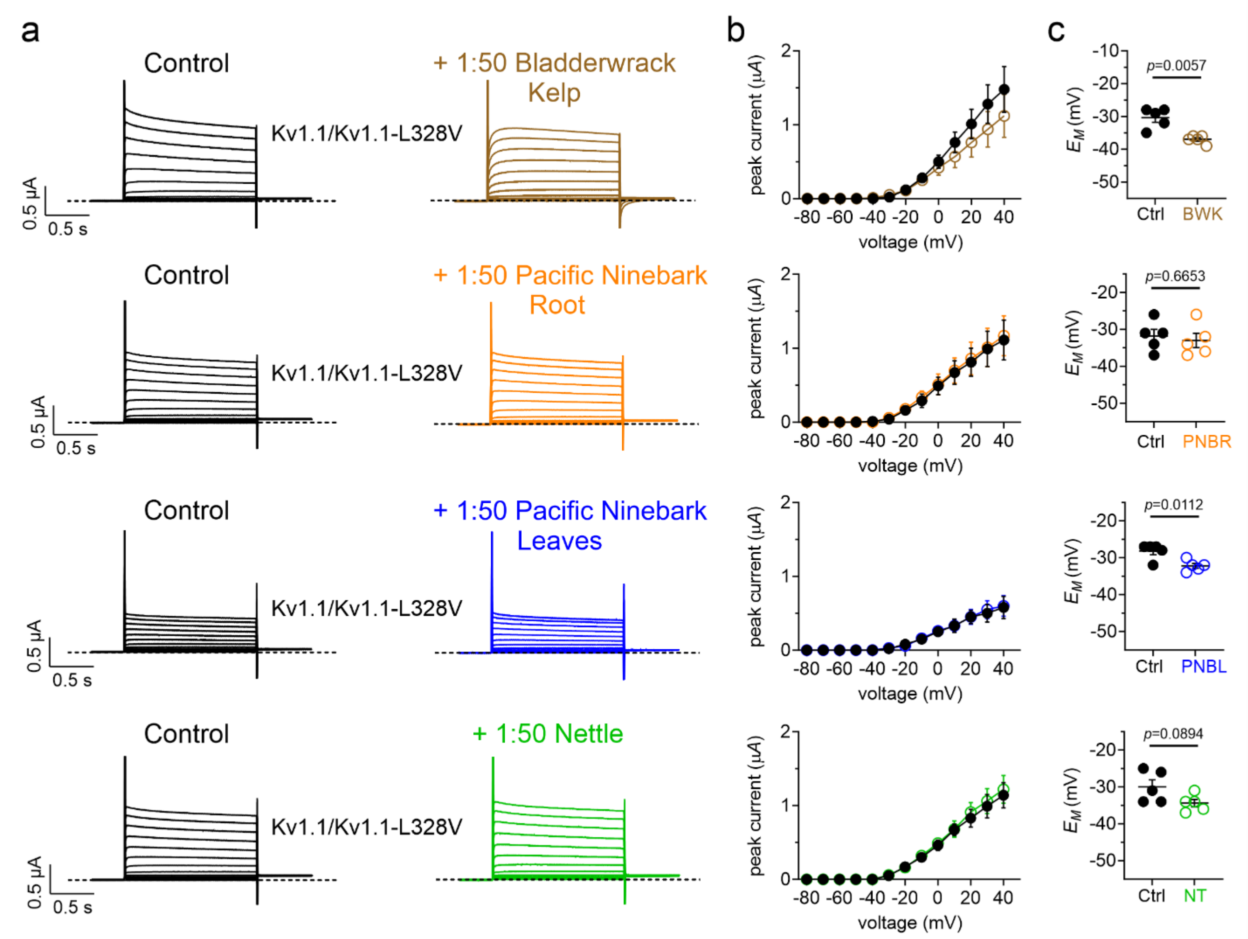

Supplementary Figure 6. Ataxia therapy plant extracts do not rescue the function of Kv1.1/Kv1.1-L328V.

Voltage protocol as in Figure 2. Error bars indicate SEM; statistical analysis by two-tailed paired t-test. At least 2 batches of oocytes were used per experiment. Magenta traces indicate same-voltage traces within each pairing for ease of visual comparison.

a. Mean trace for Kv1.1/Kv1.1-L328V in the absence (Control) and presence of plant extracts as indicated (1:50 dilution); *n* = 5.

b. Mean peak current versus voltage for Kv1.1/Kv1.1-L328V traces in a; *n* = 5.

c. Mean E_M for oocytes expressing Kv1.1/Kv1.1-L328V in the absence (Control) or presence of plant extracts as in a; bladderwrack kelp (*n* = 5; *p*=0.0057); pacific ninebark root (*n* = 5; *p*=0.6653); pacific ninebark leaves (*n* = 5; *p*=0.0112); nettle (*n* = 5; *p*=0.0894).

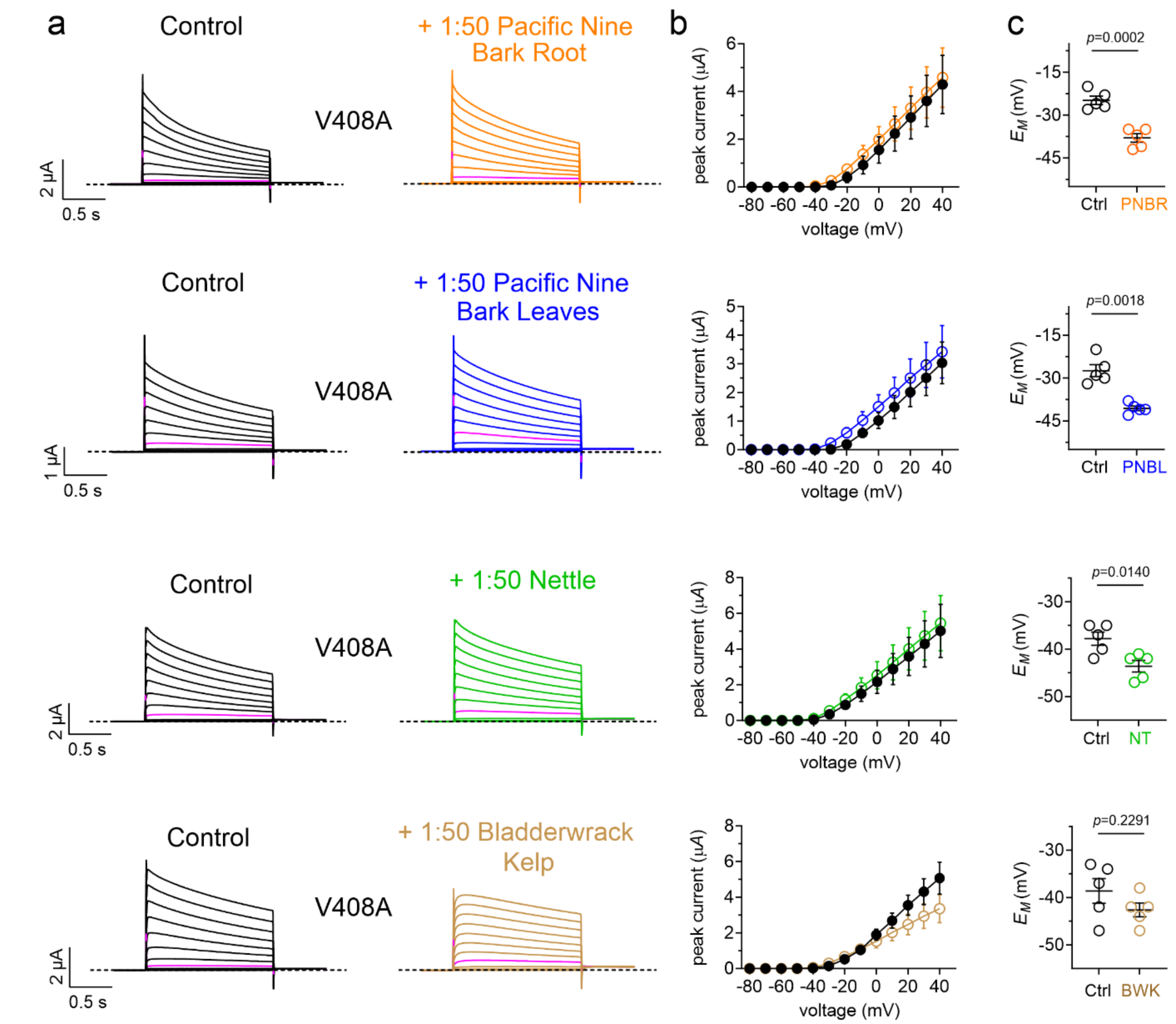

Supplementary Figure 7. Ataxia therapy plant extracts do not rescue the function of Kv1.1-V408A. Voltage protocol as in Figure 2. Error bars indicate SEM; statistical analysis by two-tailed paired t-test. At least 2 batches of oocytes were used per experiment. Magenta traces indicate same-voltage traces within each pairing for ease of visual comparison.

a. Mean trace for Kv1.1-V408A in the absence (Control) and presence of plant extracts as indicated (1:50 dilution); n = 5.

b. Mean peak current versus voltage for Kv1.1-V408A traces in a; n = 5.

c. Mean E_M for oocytes expressing Kv1.1-V408A in the absence (Control) or presence of plant extracts as in a; pacific ninebark root (n = 5; p=0.0002); pacific ninebark leaves (n = 5; p=0.0018); nettle (n = 5; p=0.0140); bladderwrack kelp (n = 5; p=0.2291).

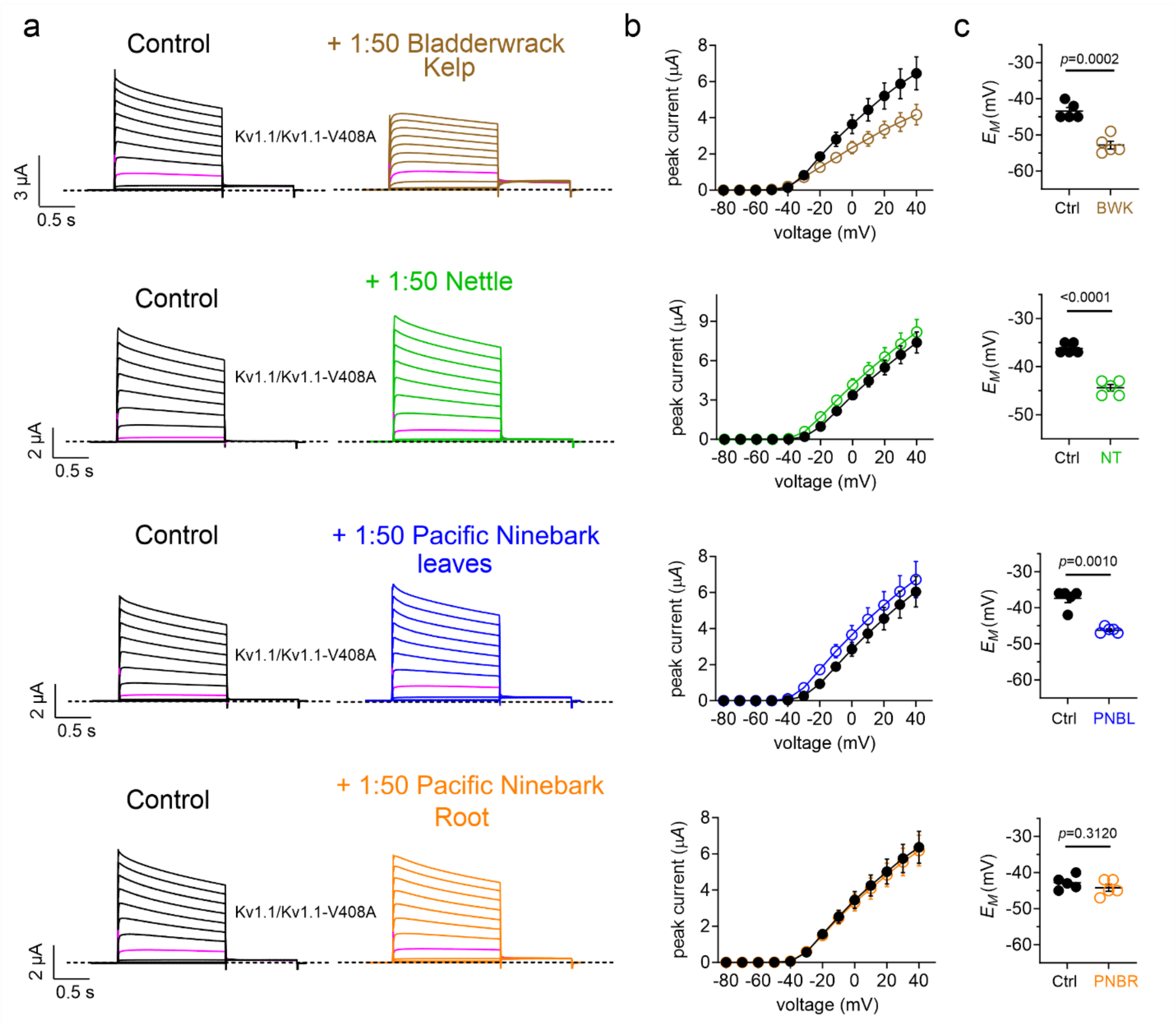

Supplementary Figure 8. Ataxia therapy plant extracts do not rescue the function of Kv1.1/Kv1.1-V408A.

Voltage protocol as in Figure 2. Error bars indicate SEM; statistical analysis by two-tailed paired t-test. At least 2 batches of oocytes were used per experiment. Magenta traces indicate same-voltage traces within each pairing for ease of visual comparison.

a. Mean trace for Kv1.1/Kv1.1-V408A in the absence (Control) and presence of plant extracts as indicated (1:50 dilution); *n* = 5.

b. Mean peak current versus voltage for Kv1.1/Kv1.1-V408A traces in a; *n* = 5.

c. Mean E_M for oocytes expressing Kv1.1/Kv1.1-V408A in the absence (Control) or presence of plant extracts as in a; bladderwrack kelp (*n* = 5; *p*=0.0002); nettle (*n* = 5; <0.0001); pacific ninebark leaves (*n* = 5; *p*=0.0010); pacific ninebark root (*n* = 5; *p*=0.3120).

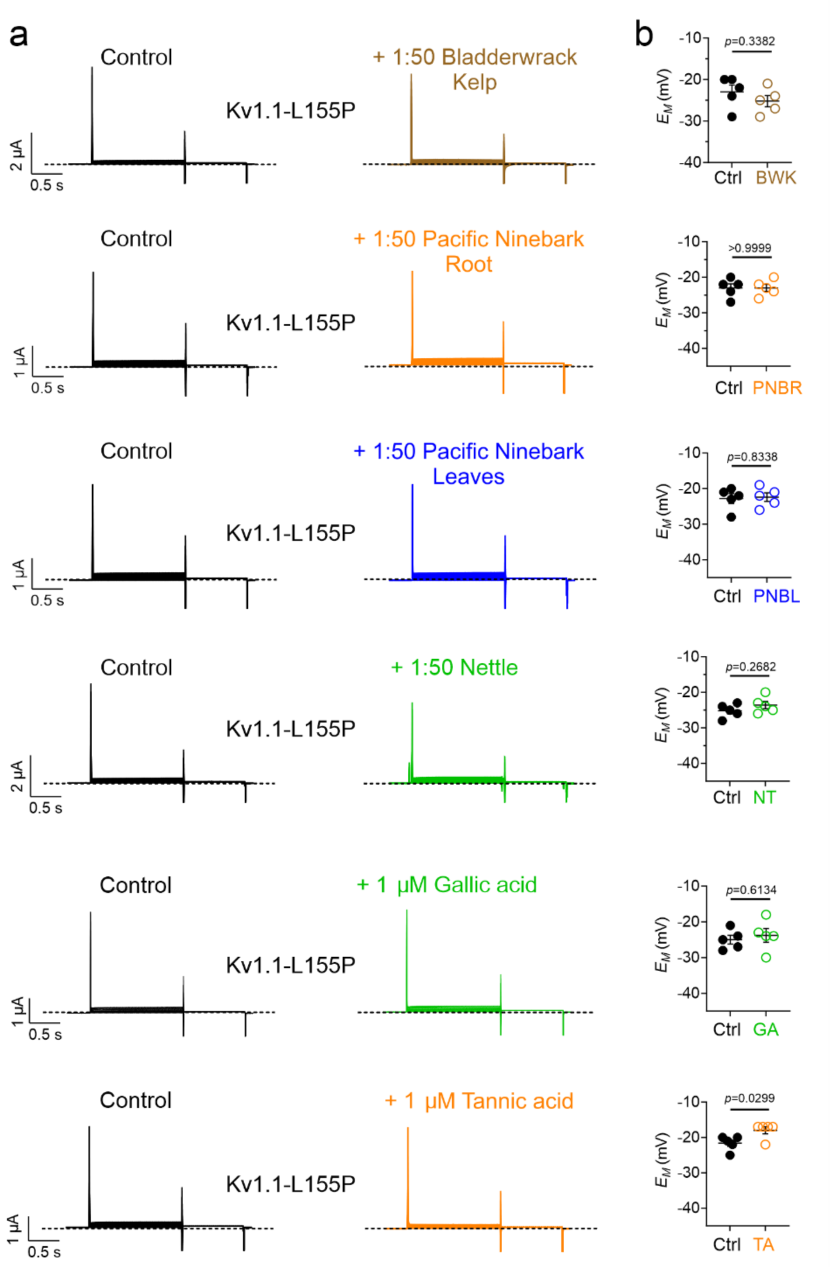

Supplementary Figure 9. Neither ataxia therapy plant extracts, nor gallic or tannic acids, rescue the function of "homozygous" Kv1.1-L155P.

Voltage protocol as in Figure 2. Error bars indicate SEM; statistical analysis by two-tailed paired t-test. At least 2 batches of oocytes were used per experiment. Magenta traces indicate same-voltage traces within each pairing for ease of visual comparison.

a. Mean trace for Kv1.1-L155P in the absence (Control) and presence of plant extracts as indicated (1:50 dilution); $n = 5$.

b. Mean E_M for oocytes expressing Kv1.1-L155P in the absence (Control) or presence of plant extracts as in a; bladderwrack kelp ($n = 5$; p=0.3382); pacific ninebark root ($n = 5$; >0.9999); pacific ninebark leaves ($n = 5$; p=0.8338); nettle ($n = 5$; p=0.2682); 1 μM gallic acid ($n = 5$; p=0.6134); 1 μM tannic acid ($n = 5$; p=0.0299).

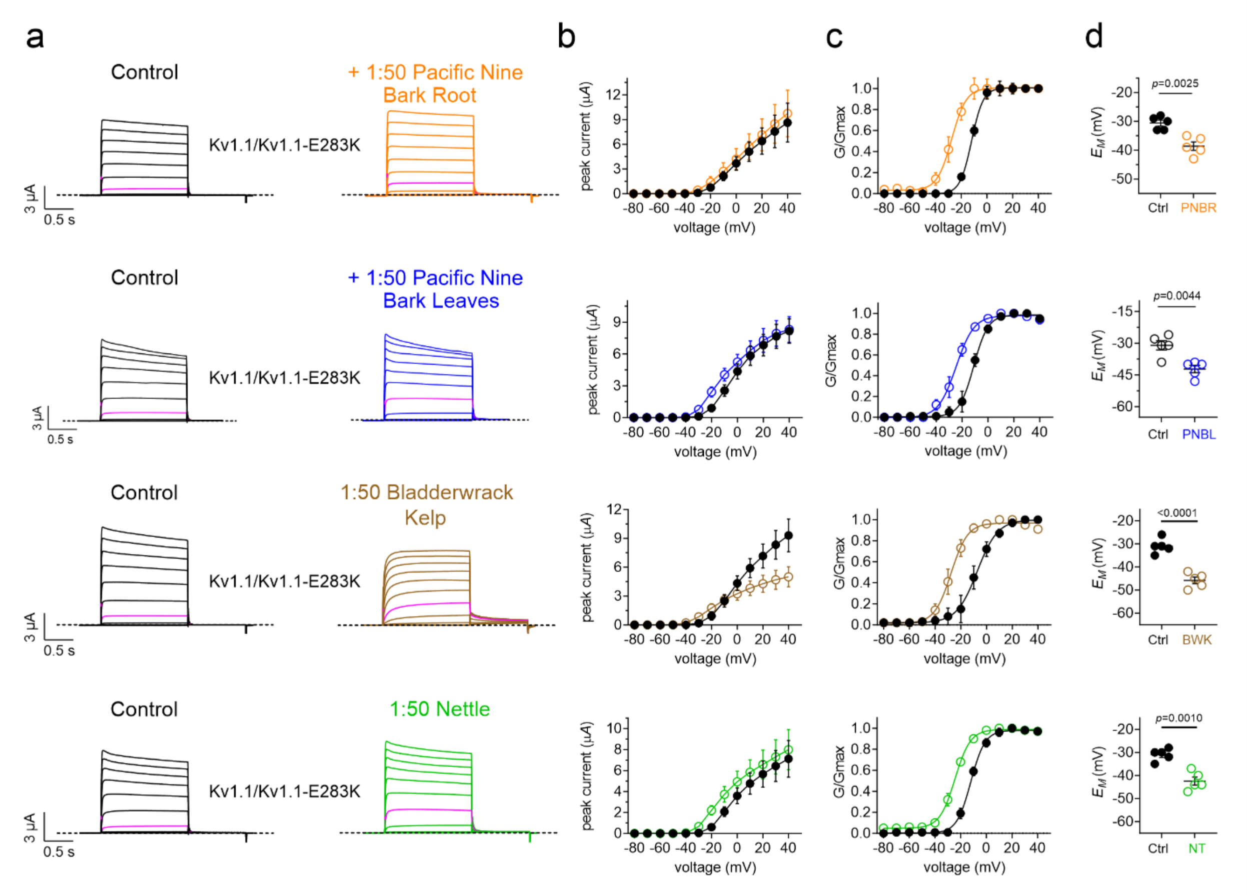

Supplementary Figure 10. Ataxia therapy plant extracts rescue the function of Kv1.1/Kv1.1-E283K. Voltage protocol as in Figure 2. Error bars indicate SEM; statistical analysis by two-tailed paired t-test. At least 2 batches of oocytes were used per experiment. Magenta traces indicate same-voltage traces within each pairing for ease of visual comparison.

a. Mean trace for Kv1.1/Kv1.1-E283K in the absence (Control) and presence of plant extracts as indicated (1:50 dilution); *n* = 5.

b. Mean peak current versus voltage for Kv1.1/Kv1.1-E283K traces in a; *n* = 5.

c. Mean G/Gmax quantified from tail current for Kv1.1/Kv1.1-E283K traces as in a; *n* = 5.

d. Mean E_M for oocytes expressing Kv1.1/Kv1.1-E283K in the absence (Control) or presence of plant extracts as in a: pacific ninebark root (*n* = 5; *p*=0.0025); pacific ninebark leaves (*n* = 5; *p*=0.0044); bladderwrack kelp (*n* = 5; <0.0001); nettle (*n* = 5; *p*=0.0010).

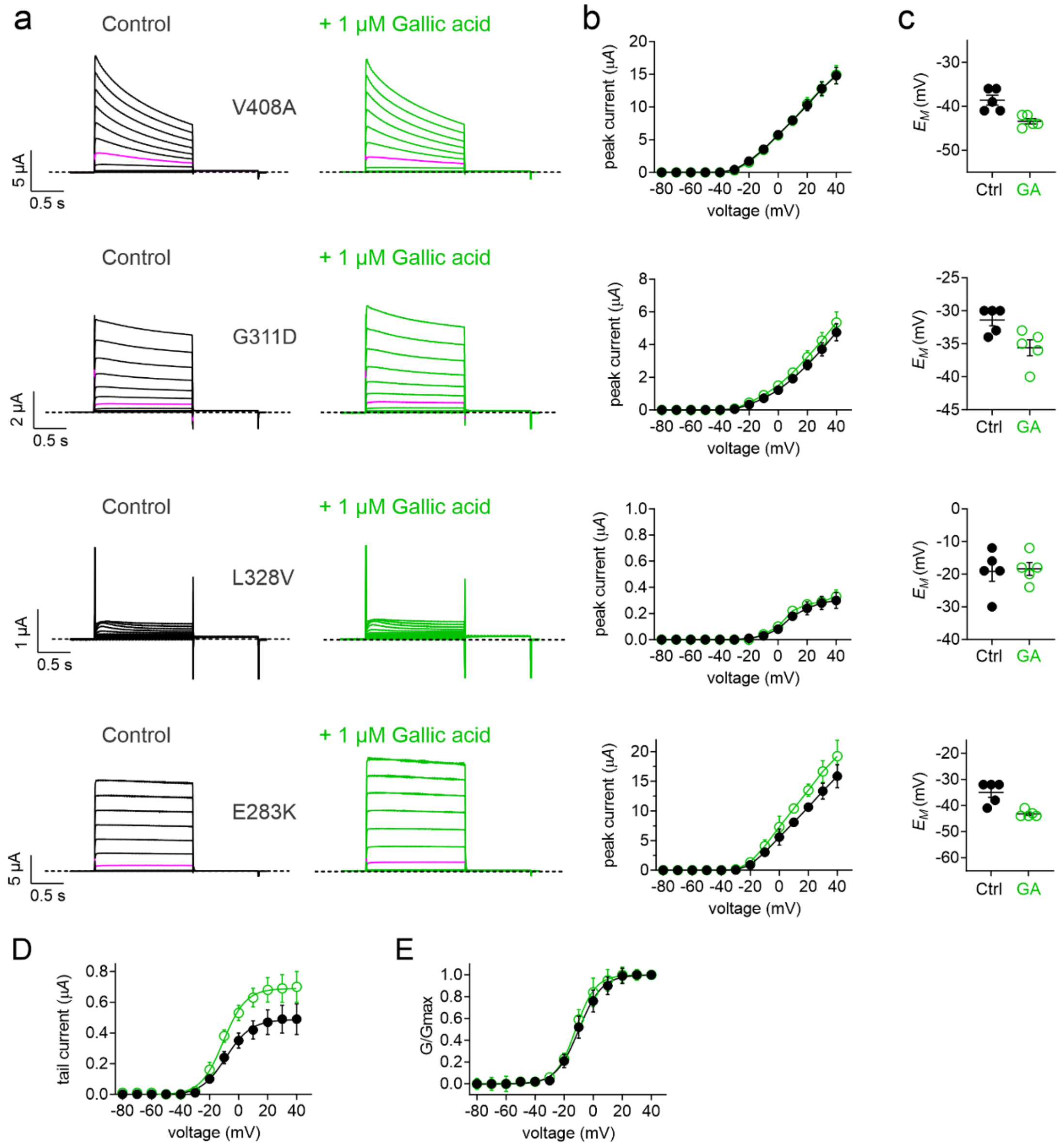

Supplementary Figure 11. Gallic acid (1 µM) is ineffective at rescuing the function of "homozygous" Kv1.1 ataxia mutant channels.

Voltage protocol as in Figure 2. Error bars indicate SEM; statistical analysis by two-tailed paired t-test. At least 2 batches of oocytes were used per experiment. Magenta traces indicate same-voltage traces within each pairing for ease of visual comparison.

a. Mean traces for ataxia mutant Kv1.1 channels as indicated in the absence (Control) and presence of gallic acid (1 µM); *n* = 5.

b. Mean peak current versus voltage for ataxia mutant Kv1.1 channels as in a; *n* = 5.

c. Mean E_M for oocytes expressing ataxia mutant Kv1.1 channels in the absence (Control) or presence of plant extracts as in a; Kv1.1-V408A (*n* = 5; p=0.0090); Kv1.1-G311D (*n* = 5; p=0.0248); Kv1.1-L328V (*n* = 5; p=0.8289); Kv1.1-E283K (*n* = 5; p=0.0101).

d. Mean tail current versus voltage for Kv1.1-E283K channels as in a; *n* = 5.

e. Mean G/Gmax versus voltage for Kv1.1-E283K channels as in a; *n* = 5.

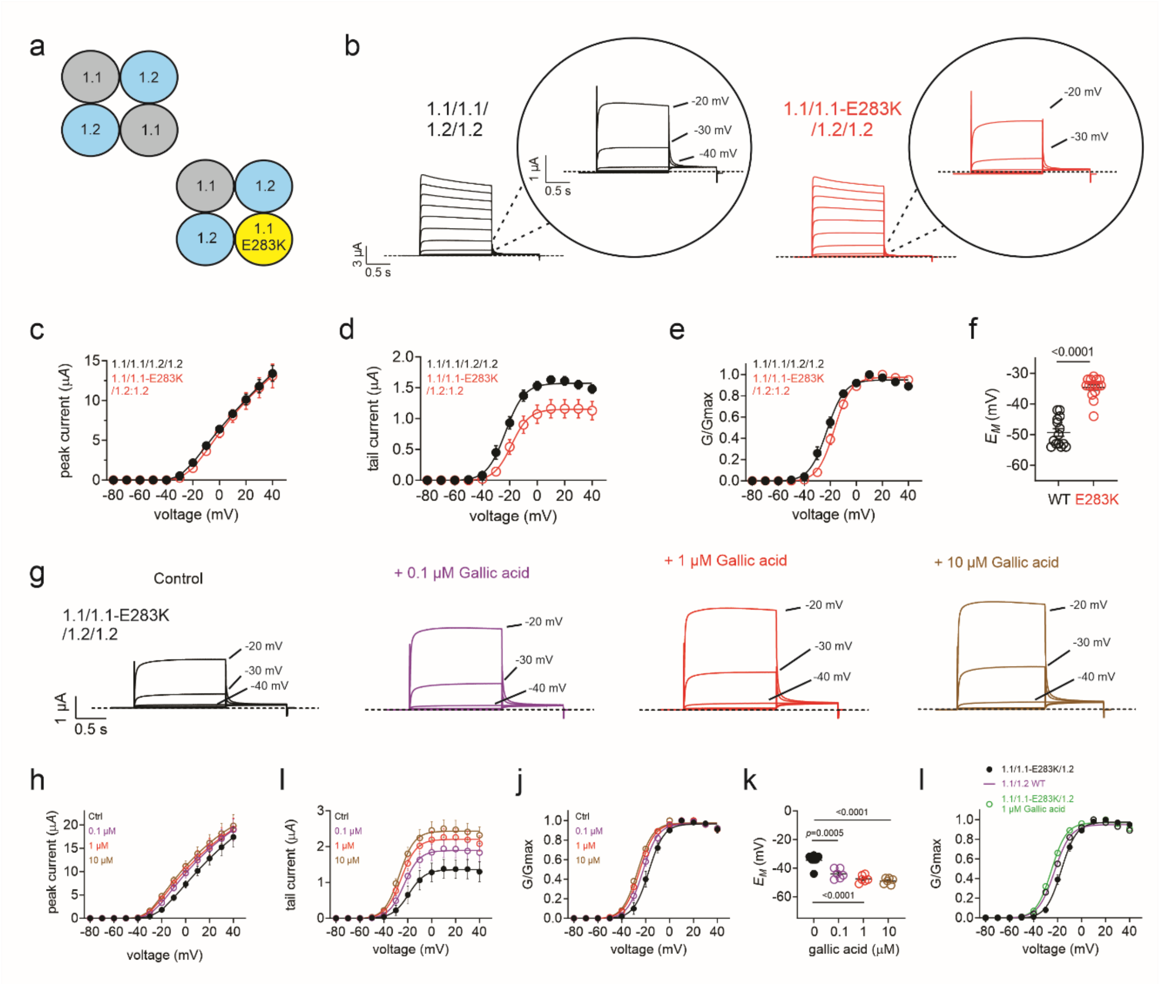

Supplementary Figure 12. Gallic acid rescues the function of EA1-linked E283K heteromeric Kv1.1-Kv1.2 channels.

Voltage protocol as in Figure 2. Error bars indicate SEM; statistical analysis by two-tailed paired t-test or One-Way ANOVA. At least 2 batches of oocytes were used per experiment.

a. cartoon representing the ratios of Kv1.x cRNA injected into each oocyte.

b. Mean traces for heteromeric wild-type (left; *n* = 14) and E283K (right; *n* = 15) Kv1.1/Kv1.2 channels expressed in oocytes; scale bars lower left. Bubbles indicate vertical scale expanded region to show reduced current in mutant channels at mildly depolarized potentials.

c-e. Mean peak, tail and normalized tail (G/Gmax) currents versus voltage for heteromeric wild-type (left; *n* = 14) and E283K (right; *n* = 15) Kv1.1/Kv1.2 channels.

f. Mean E_M for oocytes expressing heteromeric wild-type (left; *n* = 14) and E283K (right; *n* = 15) Kv1.1/Kv1.2 channels (<0.0001).

g. Mean current traces for Kv1.1/Kv1.1-E283K/Kv1.2 channels in the absence or presence of gallic acid doses as indicated (*n* = 6).

h-j. Mean peak, tail, and normalized (G/Gmax) currents versus voltage for channels as in g; *n* = 6.

k. Mean E_M for oocytes expressing Kv1.1/Kv1.1-E283K/Kv1.2 channels in; 0.1 µM gallic acid (*n* = 6; *p*=0.0005); 1 µM gallic acid (*n* = 6; <0.0001); 10 µM gallic acid (*n* = 6; <0.0001).

l. Comparison of mean normalized tail currents (G/Gmax) showing that gallic acid (1 µM) returns mutant E283K Kv1.1/Kv1.2 (*n* = 6) channel voltage dependence to match that heteromeric wild type (*n* = 14).

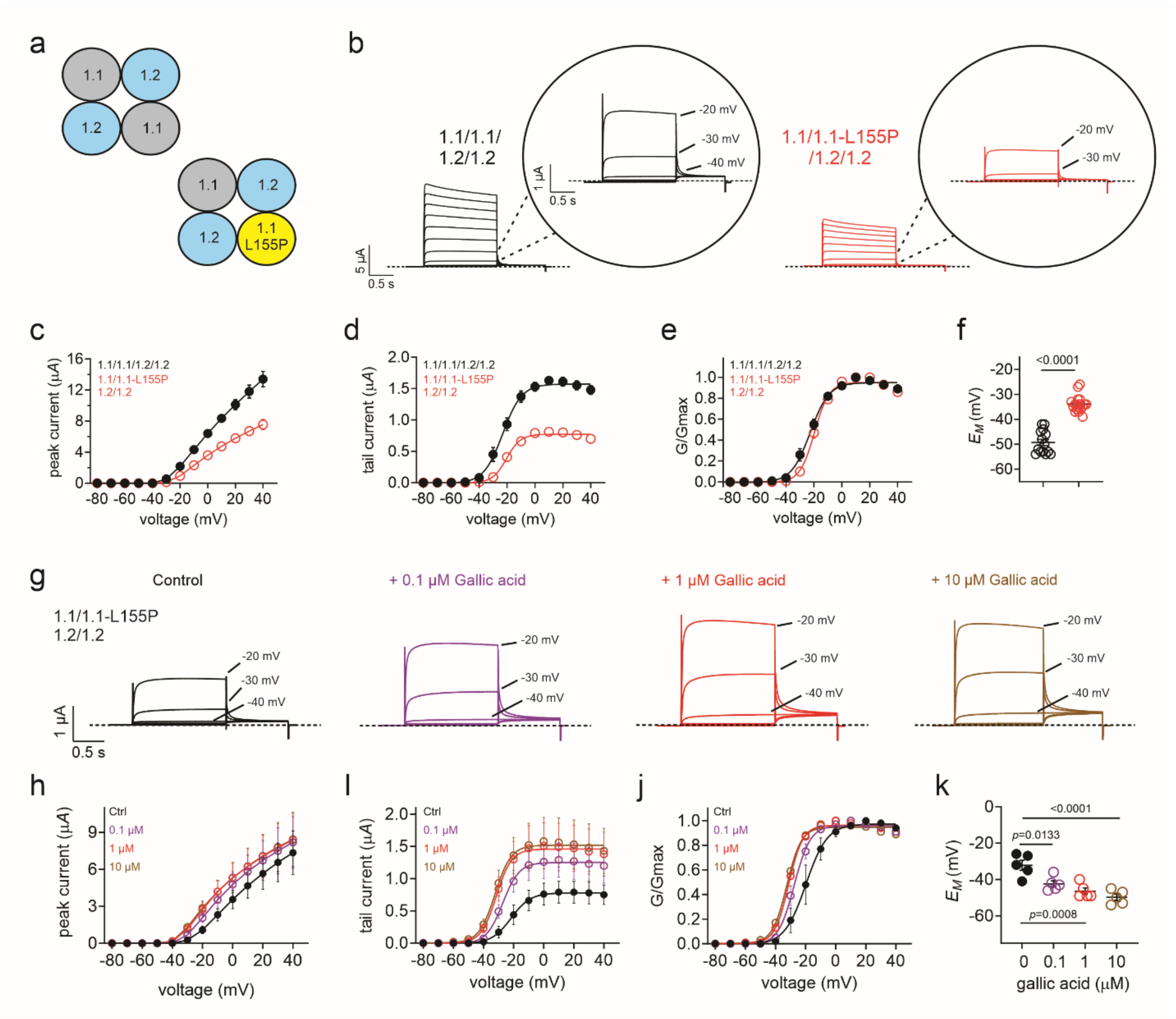

Supplementary Figure 13. Gallic acid rescues the function of EA1-linked L155P heteromeric Kv1.1-Kv1.2 channels.

Voltage protocol as in Figure 2. Error bars indicate SEM; statistical analysis by two-tailed paired t-test or One-Way ANOVA. At least 2 batches of oocytes were used per experiment.

a. cartoon representing the ratios of Kv1.x cRNA injected into each oocyte.

b. Mean traces for heteromeric wild-type (left; *n* = 14) and L155P (right; *n* = 18) Kv1.1/Kv1.2 channels expressed in oocytes; scale bars lower left. Bubbles indicate vertical scale expanded region to show reduced current in mutant channels at mildly depolarized potentials.

c-e. Mean peak, tail, and normalized tail (G/Gmax) currents versus voltage for heteromeric wild-type (left; *n* = 14) and L155P (right; *n* = 18) Kv1.1/Kv1.2 channels as in b.

f. Mean E_M for oocytes expressing heteromeric wild-type (left; *n* = 14) and L155P (right; *n* = 18) Kv1.1/Kv1.2 channels as in b (<0.0001).

g. Mean current traces for Kv1.1/Kv1.1-L155P/Kv1.2 channels in the absence or presence of gallic acid doses as indicated (*n* = 5).

h-j. Mean peak, tail, and normalized (G/Gmax) currents versus voltage for channels as in g; *n* = 5.

k. Mean E_M for oocytes expressing Kv1.1/Kv1.1-L155P/Kv1.2 channels in; 0.1 µM gallic acid (*n* = 5; *p*=0.0133); 1 µM gallic acid (*n* = 5; *p*=0.0008); 10 µM gallic acid (*n* = 5; <0.0001).

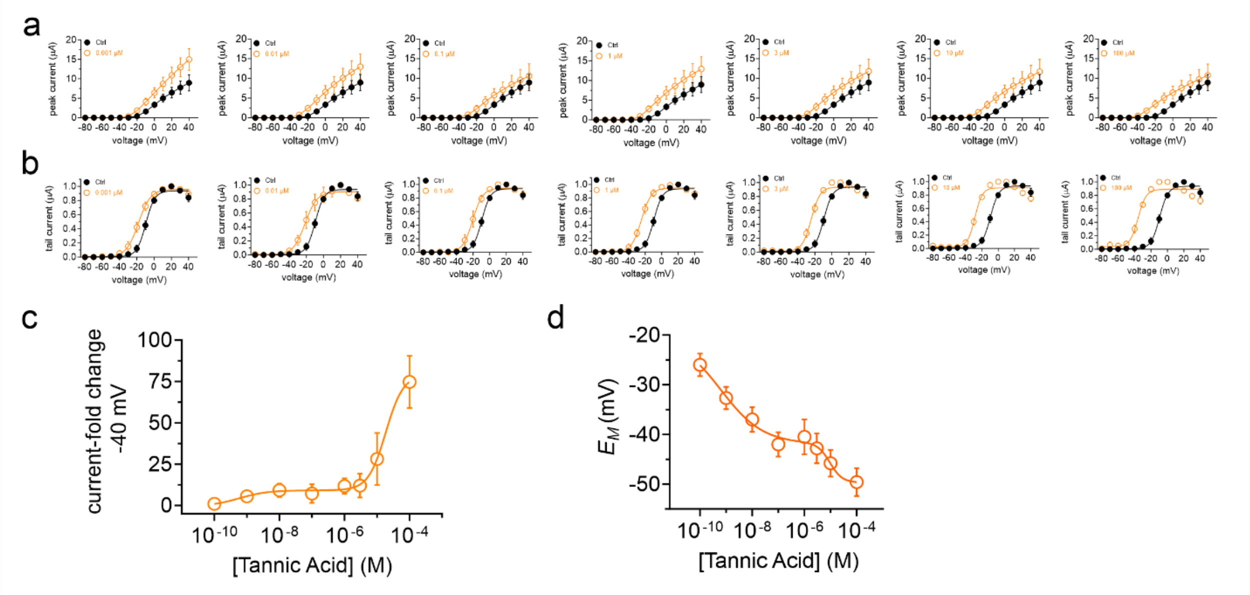

Supplementary Figure 14. Dose response for tannic acid effects on Kv1.1-E283K.

Voltage protocol as in Figure 2. Error bars indicate SEM. At least 2 batches of oocytes were used per experiment.

a. Mean peak current versus voltage for Kv1.1-E283K in the absence (Control) or presence of tannic acid concentrations: 0.001 μM (*n* = 4); 0.01 μM (*n* = 4); 0.1 μM (*n* = 6); 1 μM (*n* = 4); 3 μM (*n* = 6); 10 μM (*n* = 6); 100 μM (*n* = 6).

b. Mean G/Gmax versus voltage for Kv1.1-E283K in the absence (Control) or presence of tannic acid concentrations as indicated: 0.001 μM (*n* = 4); 0.01 μM (*n* = 4); 0.1 μM (*n* = 6); 1 μM (*n* = 4); 3 μM (*n* = 6); 10 μM (*n* = 6); 100 μM (*n* = 6).

c. Dose response for tannic acid effects at -40 mV on Kv1.1-E283K calculated from graphs as in A. 0.001 μM (*n* = 3); 0.01 μM (*n* = 4); 0.1 μM (*n* = 6); 1 μM (*n* = 4); 3 μM (*n* = 6); 10 μM (*n* = 6); 100 μM (*n* = 6).

d. Dose response for tannic acid effects on E_M of oocytes expressing Kv1.1-E283K, calculated from as in a: 0.001 μM (*n* = 4); 0.01 μM (*n* = 4); 0.1 μM (*n* = 6); 1 μM (*n* = 4); 3 μM (*n* = 6); 10 μM (*n* = 6); 100 μM (*n* = 6).

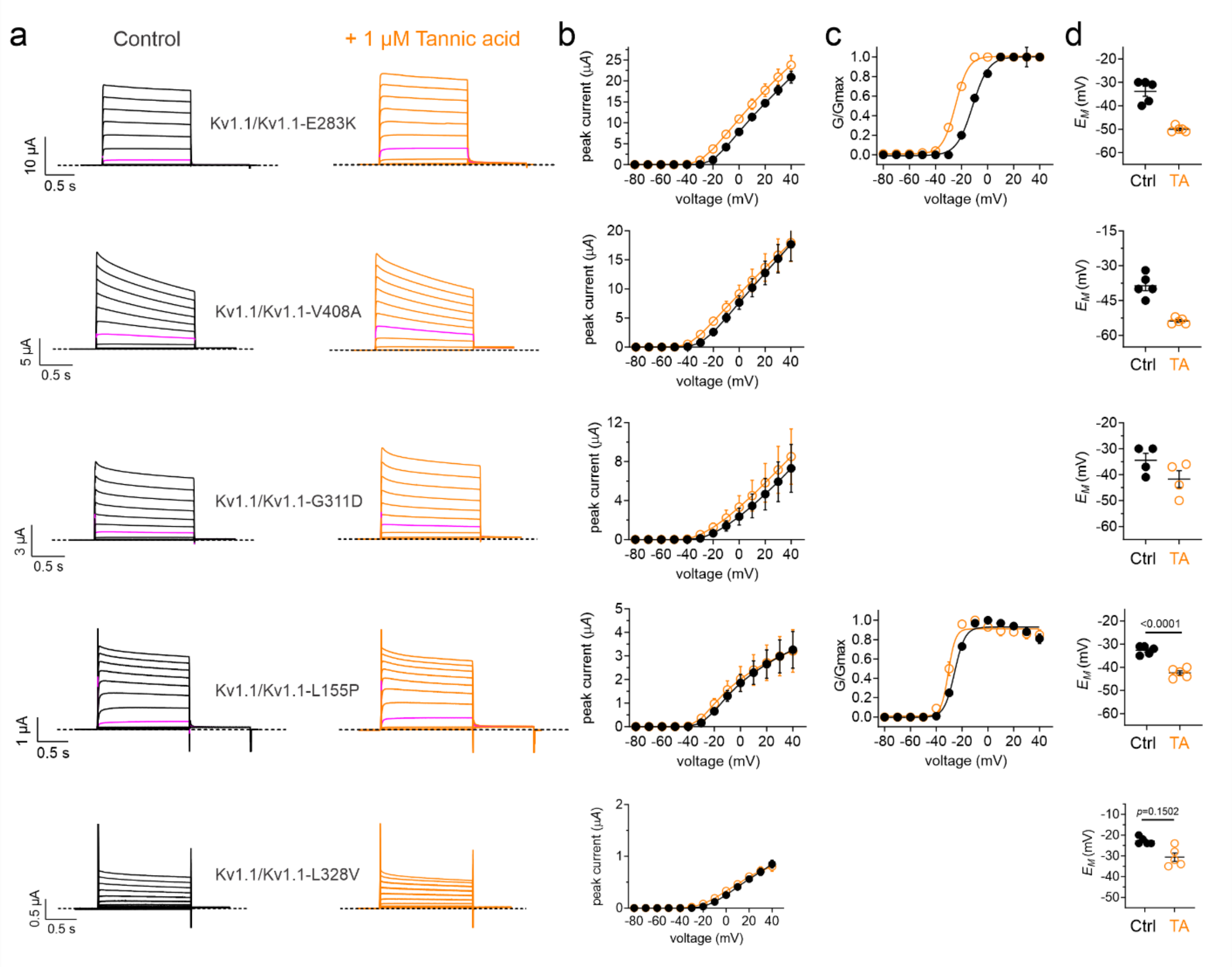

Supplementary Figure 15. Tannic acid (1 µM) enhances Kv1.1/Kv1.1-E283K but no other mixed wild-type/ataxia mutant Kv1.1 channels.

Voltage protocol as in Figure 2. Error bars indicate SEM; statistical analysis by two-tailed paired t-test. At least 2 batches of oocytes were used per experiment. Magenta traces indicate same-voltage traces within each pairing for ease of visual comparison.

a. Mean trace for heteromeric channels as indicated in the absence (Control) and presence of tannic acid (1 µM): Kv1.1/Kv1.1-E283K (*n* = 5); Kv1.1/Kv1.1-V408A (*n* = 5); Kv1.1/Kv1.1-G311D (*n* = 4); Kv1.1/Kv1.1-L155P (*n* = 5); Kv1.1/Kv1.1-L328V (*n* = 5).

b. Mean peak currents versus voltage for traces as in a: Kv1.1/Kv1.1-E283K (*n* = 5); Kv1.1/Kv1.1-V408A (*n* = 5); Kv1.1/Kv1.1-G311D (*n* = 4); Kv1.1/Kv1.1-L155P (*n* = 5); Kv1.1/Kv1.1-L328V (*n* = 5).

c. Mean G/Gmax versus voltage for traces as in a: Kv1.1/Kv1.1-E283K (*n* = 5); Kv1.1/Kv1.1-V408A (*n* = 5); Kv1.1/Kv1.1-G311D (*n* = 4); Kv1.1/Kv1.1-L155P (*n* = 5); Kv1.1/Kv1.1-L328V (*n* = 5). Graphs omitted where tail currents were too small to quantify.

d. Mean E_M for oocytes expressing channels as in a in the absence (Control) or presence of tannic acid (1 µM) Kv1.1/Kv1.1-E283K (*n* = 5; *p*=0.0012); Kv1.1/Kv1.1-V408A (*n* = 5; *p*=0.0016); Kv1.1/Kv1.1-G311D (*n* = 4; *p*=0.1413); Kv1.1/Kv1.1-L155P (*n* = 5; <0.0001); Kv1.1/Kv1.1-L328V (*n* = 5; *p*=0.1502).

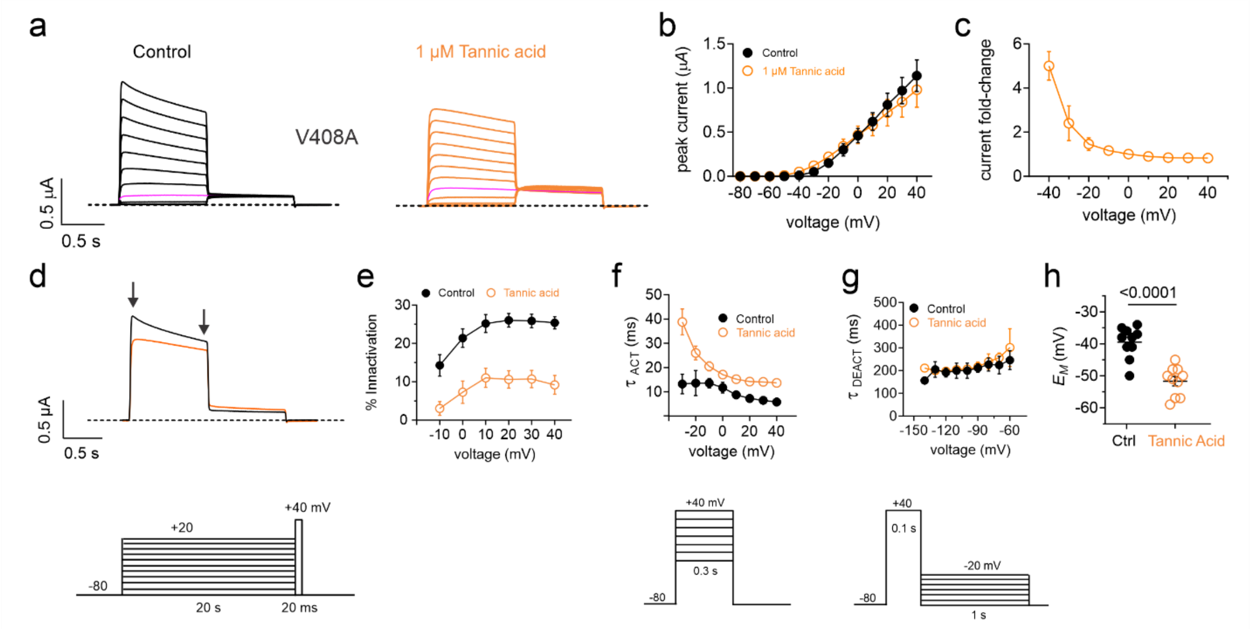

Supplementary Figure 16. Tannic acid (1 µM) effects on Kv1.1-V408A channels.

Error bars indicate SEM; statistical analysis by two-tailed paired t-test. At least 2 batches of oocytes were used per experiment. Magenta traces indicate same-voltage traces within each pairing for ease of visual comparison.

a. Mean trace for homomeric Kv1.1-V408A channels in the absence (Control) and presence of tannic acid (1 µM); *n* = 10; Voltage protocols as in Figure 2.

b. Mean peak current versus voltage for traces as in a; *n* = 10.

c. Current fold change induced by (1 µM) tannic acid versus voltage for traces as in a; *n* = 10.

d. Mean traces showing effects of tannic acid (1 µM) on Kv1.1-V408A inactivation (between the two vertical bars) quantified using the voltage protocol shown (lower inset); *n* = 10.

e. Effects of tannic acid (1 µM) on % inactivation quantified as in d; *n* = 10.

f. Mean activation rate (T_{ACT}) versus voltage for Kv1.1-V408A in bath solution (black) versus tannic acid (1 µM) (brown), quantified using the voltage protocol shown (lower inset); *n* = 10.

g. Mean deactivation rate (T_{DEACT}) versus voltage for Kv1.1-V408A in bath solution (black) versus tannic acid (1 µM) (brown), quantified using the voltage protocol shown (lower inset); *n* = 8.

h. Mean E_M for oocytes expressing channels as in a in the absence (Control) or presence of tannic acid (1 µM); (*n* = 10; <0.0001).

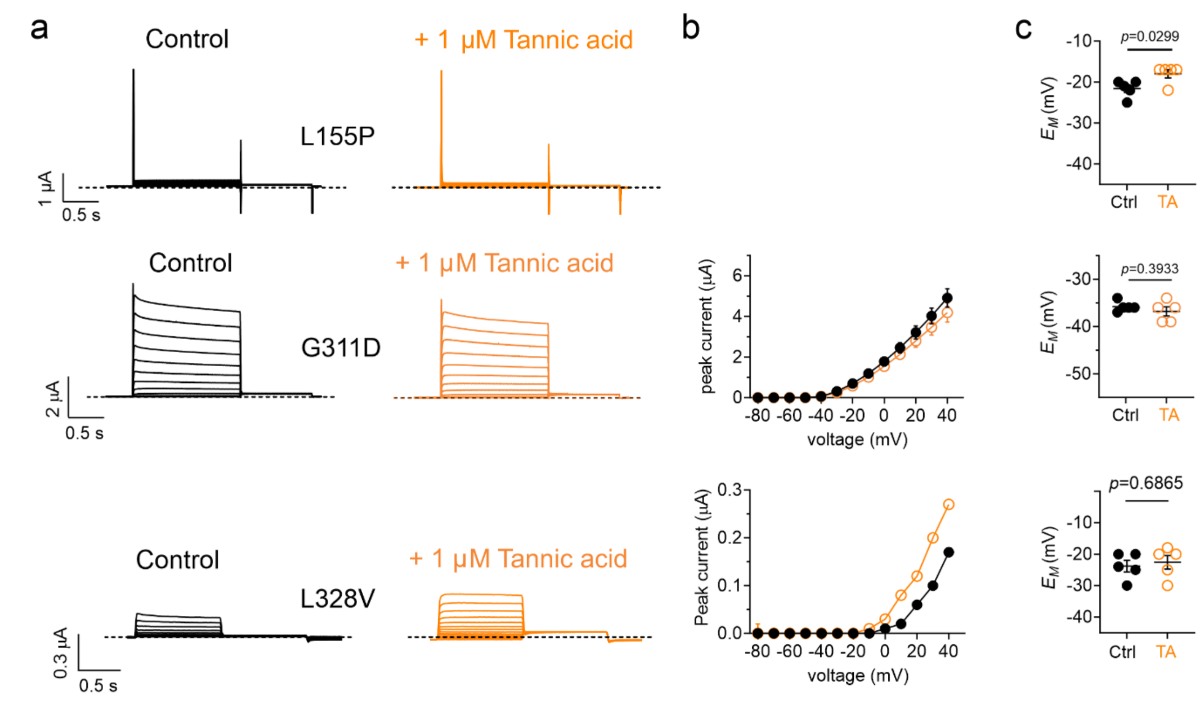

Supplementary Figure 17. Tannic acid (1 µM) does not rescue 100% mutant L155P, G311D or L328V Kv1.1 activity.

Voltage protocol as in Figure 2. Error bars indicate SEM; statistical analysis by two-tailed paired t-test. At least 2 batches of oocytes were used per experiment.

a. Mean trace for channel as indicated in the absence (Control) and presence of tannic acid (1 µM); n = 5.

b. Mean peak current versus voltage for traces as in a; n = 5.

c. Mean E_M for oocytes expressing channels as in a in the absence (Control) and presence Tannic acid (1 µM): Kv1.1-L155P (n = 5; p=0.0299); Kv1.1-G311D (n = 5; p=0.3933); Kv1.1-L328V (n = 5; p=0.6865).

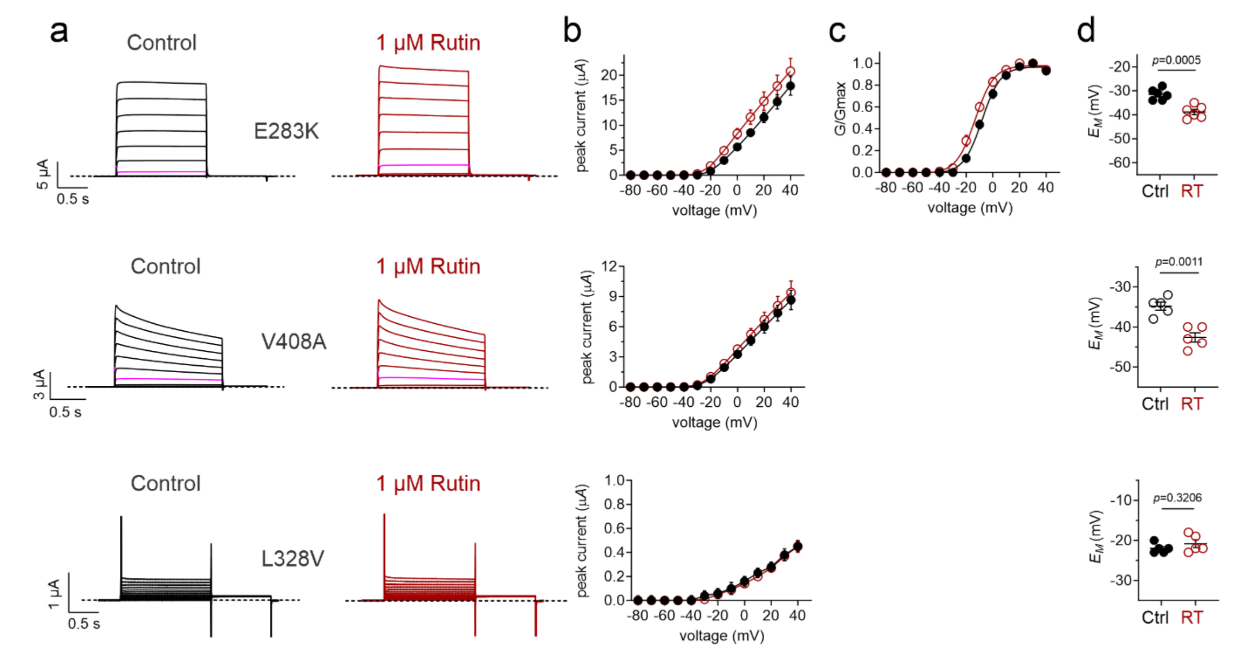

Supplementary Figure 18. Rutin (1 µM) is ineffective at enhancing ataxia mutant Kv1.1 channel activity.

Voltage protocol as in Figure 2. Error bars indicate SEM; statistical analysis by two-tailed paired t-test. At least 2 batches of oocytes were used per experiment. Magenta traces indicate same-voltage traces within each pairing for ease of visual comparison.

a. Mean trace for channels as indicated in the absence (Control) and presence of rutin (1 µM): Kv1.1-E283K (n = 6); Kv1.1-V408A (n = 5); Kv1.1-L328V (n = 5).

b. Mean peak current versus voltage for traces as in a: Kv1.1-E283K (n = 6); Kv1.1-V408A (n = 5); Kv1.1-L328V (n = 5).

c. Mean G/Gmax versus voltage for traces as in a: Kv1.1-E283K (n = 6); Kv1.1-V408A (n = 5); Kv1.1-L328V (n = 5). Graphs omitted where tail currents were too small to quantify.

d. Mean E_M for oocytes expressing channels as in a in the absence (Control) and presence of rutin (1 µM); Kv1.1-E283K (n = 6; p=0.0005); Kv1.1-V408A (n = 5; p=0.0011); Kv1.1-L328V (n = 5; p=0.3206).

Supplementary Data – values and statistics tabulated by figure number.

Figure 1

	$V_{0.5}$ Normalized tail current (mV)	Slope (mV)	E_M (mV)
Control	-27.13 ± 1.47	5.56 ± 1.47	-39.80 ± 1.73
1:50 Nettle	-42.46 ± 1.94 (*p*=0.0003; n=5)	7.46 ± 1.69 (*p*=0.4214; n=5)	-45.40 ± 1.23 (*p*=0.0211; n=5)

Statistics versus Kv1.1 in absence of Nettle. Values indicate mean ± SEM.

	$V_{0.5}$ Normalized tail current (mV)	Slope (mV)	E_M (mV)
Control	-26.02 ± 1.15	5.93 ± 0.98	-41.20 ± 1.40
1:50 Pacific Ninebark Root	-38.85 ± 1.31 (<0.0001; n=5)	4.48 ± 1.25 (*p*=0.3895; n=5)	-51.80 ± 1.97 (*p*=0.0028; n=5)

Statistics versus Kv1.1 in absence of Pacific Ninebark Root. Values indicate mean ± SEM.

	$V_{0.5}$ Normalized tail current (mV)	Slope (mV)	E_M (mV)
Control	-25.42 ± 1.29	6.45 ± 1.12	-47.40 ± 2.00
1:50 Pacific Ninebark Leaves	-36.56 ± 1.19 (*p*=0.0002; n=5)	6.39 ± 1.04 (*p*=0.9590; n=5)	-53.60 ± 1.34 (*p*=0.0201; n=5)

Statistics versus Kv1.1 in absence of Pacific Ninebark Leaves. Values indicate mean ± SEM.

	$V_{0.5}$ Normalized tail current (mV)	Slope (mV)	E_M (mV)
Control	-24.39 ± 0.77	6.76 ± 0.67	-36.80 ± 1.36
1:50 Bladderwrack Kelp	-41.82 ± 1.02 (<0.0001; n=5)	3.30 ± 1.04 (*p*=0.0273; n=5)	-44.80 ± 1.34 (*p*=0.0097; n=5)

Statistics versus Kv1.1 in absence of Bladderwrack Kelp. Values indicate mean ± SEM.

	$V_{0.5}$ Normalized tail current (mV)	Slope (mV)	E_M (mV)
Control	-16.86 ± 0.38	4.97 ± 0.33	-30.50 ± 1.41
1:50 Nettle	-30.23 ± 0.71 (<0.0001; n=5)	4.36 ± 0.71 (*p*=0.4673; n=5)	-44.67 ± 2.41 (*p*=0.0007; n=5)

Statistics versus Kv1.2 in absence of Nettle. Values indicate mean ± SEM.

	$V_{0.5}$ Normalized tail current (mV)	Slope (mV)	E_M (mV)
Control	-25.04 ± 0.45	6.45 ± 0.39	-39.60 ± 1.56
1:50 Pacific Ninebark Root	-27.28 ± 0.58 (*p*=0.0170; n=5)	6.13 ± 0.50 (*p*=0.6282; n=5)	-42.60 ± 0.61 (*p*=0.0915; n=5)

Statistics versus Kv1.2 in absence of Pacific Ninebark Root. Values indicate mean ± SEM.

	$V_{0.5}$ Normalized tail current (mV)	Slope (mV)	E_M (mV)
Control	-28.60 ± 0.40	7.46 ± 0.35	-48.00 ± 2.05
1:50 Pacific Ninebark Leaves	-28.31 ± 0.46 (*p*=0.6472; n=5)	7.17 ± 0.41 (*p*=0.6056; n=5)	-46.20 ± 1.80 (*p*=0.4156; n=5)

Statistics versus Kv1.2 in absence of Pacific Ninebark Leaves. Values indicate mean ± SEM.

	$V_{0.5}$ Normalized tail current (mV)	Slope (mV)	E_M (mV)
Control	-19.95± 0.83	6.40 ± 0.73	-33.67 ± 1.20
1:50 Bladderwrack Kelp	-34.48 ± 2.13 (*p*=0.0005; n=6)	6.86 ± 1.86 (*p*=0.83; n=6)	-48.67 ± 0.7 (*p*=0.0009; n=6)

Statistics versus Kv1.2 in absence of Bladderwrack Kelp. Values indicate mean ± SEM.

Figure 2

	$V_{0.5}$ Normalized tail current (mV)	Slope (mV)	E_M (mV)
Control	-11.67 ± 0.96	6.56 ± 0.84	-29.33 ± 1.92
100 µM Catechin Hydrate	-14.70 ± 0.44 (*p*=0.0242; n=6)	5.83 ± 0.38 (*p*=0.4549; n=6)	-31.33 ± 2.31 (*p*=0.3238; n=6)

Statistics versus Kv1.1 in absence of catechin hydrate. Values indicate mean ± SEM.

	$V_{0.5}$ Normalized tail current (mV)	Slope (mV)	E_M (mV)
Control	-11.55 ± 1.55	9.57 ± 1.40	-36.20 ± 0.78
100 µM Gallic acid	-37.17 ± 2.59 (<0.0001; n=5)	12.10 ± 2.32 (*p*=0.3835; n=5)	-49.60 ± 0.91 (*p*=0.0002; n=5)

Statistics versus Kv1.1 in absence of Gallic acid. Values indicate mean ± SEM.

	$V_{0.5}$ Normalized tail current (mV)	Slope (mV)	E_M (mV)
Control	-22.35 ± 0.42	6.25 ± 0.56	-40.00 ± 2.53
100 µM Cytisine	-23.96 ± 1.37 (*p*=0.3148; n=5)	8.15 ± 1.31 (*p*=0.2357; n=5)	-41.60 ± 1.77 (*p*=0.3949; n=5)

Statistics versus Kv1.1 in absence of Cytisine. Values indicate mean ± SEM.

	$V_{0.5}$ Normalized tail current (mV)	Slope (mV)	E_M (mV)
Control	-21.99 ± 0.91	9.43 ± 0.92	-45.75 ± 1.20
100 µM Kaempferol	-22.40 ± 0.84 (*p*=0.8458; n=5)	8.68 ± 0.87 (*p*=0.5700; n=5)	-45.25 ± 1.83 (*p*=0.6997; n=5)

Statistics versus Kv1.1 in absence of Kaempferol. Values indicate mean ± SEM.

	$V_{0.5}$ Normalized tail current (mV)	Slope (mV)	E_M (mV)
Control	-25.70 ± 1.83	6.61 ± 1.10	-43.75 ± 3.30
100 µM Quercetin	-25.12 ± 1.81 (*p*=0.8292; n=4)	6.00 ± 1.06 (>0.9999; n=4)	-43.50 ± 1.46 (*p*=0.9438; n=4)

Statistics versus Kv1.1 in absence of Quercetin. Values indicate mean ± SEM.

	$V_{0.5}$ Normalized tail current (mV)	Slope (mV)	E_M (mV)
Control	-22.70 ± 1.29	8.58 ± 1.28	-44.50 ± 1.73
100 µM Rutin	-31.59 ± 3.22 (*p*=0.0634; n=4)	11.79 ± 2.88 (*p*=0.3642; n=4)	-56.75 ± 0.28 (*p*=0.0065; n=4)

Statistics versus Kv1.1 in absence of Rutin. Values indicate mean ± SEM.

	$V_{0.5}$ Normalized tail current (mV)	Slope (mV)	E_M (mV)
Control	-23.92 ± 1.01	8.41 ± 0.89	-38.40 ± 1.50
100 µM Tannic acid	-44.95 ± 2.49 (*p*=0.0004; n=5)	13.67 ± 2.01 (*p*=0.0575; n=5)	-54.40 ± 1.23 (<0.0001; n=5)

Statistics versus Kv1.1 in absence of Tannic acid. Values indicate mean ± SEM.

Figure 3

	$V_{0.5}$ Normalized tail current (mV)	Slope (mV)	E_M (mV)
Control	-21.91 ± 1.77	12.21 ± 1.58	-41.40 ± 1.00
1:50 Wild Oak Bark	-38.27 ± 1.73 (*p*=0.0002; n=5)	10.44 ± 1.53 (*p*=0.4442; n=5)	-53.40 ± 0.63 (<0.0001; n=5)

Statistics versus Kv1.1 in absence of White Oak Bark. Values indicate mean ± SEM.

	$V_{0.5}$ Normalized tail current (mV)	Slope (mV)	E_M (mV)
Control	-22.59 ± 1.76	11.34 ± 1.56	-45.40 ± 1.20
1:50 Cramp Bark	-35.31 ± 1.14 (*p*=0.0006; n=5)	10.69 ± 0.74 (*p*=0.8283; n=5)	-48.20 ± 1.77 (*p*=0.4146; n=5)

Statistics versus Kv1.1 in absence of Cramp Bark. Values indicate mean ± SEM.

	$V_{0.5}$ Normalized tail current (mV)	Slope (mV)	E_M (mV)
Control	-22.19 ± 3.57	17.40 ± 3.49	-49.00 ± 1.32
1:50 Wild Cherry Bark	-36.46 ± 1.19 (*p*=0.0133; n=5)	11.43 ± 1.06 (*p*=0.1659; n=5)	-58.40 ± 0.97 (*p*=0.0346; n=5)

Statistics versus Kv1.1 in absence of Wild Cherry Bark. Values indicate mean ± SEM.

	$V_{0.5}$ Normalized tail current (mV)	Slope (mV)	E_M (mV)
Control	-21.46 ± 2.60	11.75 ± 2.31	-45.50 ± 0.91
1:50 White Willow Bark	-36.28 ± 0.83 (*p*=0.0032; n=5)	10.69 ± 0.74 (*p*=0.6810; n=5)	-57.40 ± 0.20 (<0.0001; n=5)

Statistics versus Kv1.1 in absence of White Willow Bark. Values indicate mean ± SEM.

	$V_{0.5}$ Normalized tail current (mV)	Slope (mV)	E_M (mV)
Control	-28.46 ± 1.36	8.47 ± 1.04	-48.33 ± 0.26
1:50 *Sophora Japonica*	-45.86 ± 2.43 (*p*=0.0430; n=6)	8.31 ± 2.04 (*p*=0.1863; n=6)	-65.83 ± 2.29 (*p*=0.0002; n=6)

Statistics versus Kv1.1 in absence of *Sophora Japonica*. Values indicate mean ± SEM.

	$V_{0.5}$ Normalized tail current (mV)	Slope (mV)	E_M (mV)
Control	-19.83 ± 0.65	7.93 ± 0.51	-35.12 ± 3.20
100 µM Oxymatrine	-21.86 ± 0.64 (*p*=xxx; n=8)	8.73 ± 0.24 (*p*=xxx; n=8)	-39.88 ± 2.34 (*p*=0.1720; n=8)

Statistics versus Kv1.1 in absence of Oxymatrine. Values indicate mean ± SEM.

Figure 4

KV1.1	**EC50 (nM)**
Tannic acid	136 ± 30 (n=7-12)
Gallic acid	379 ± 28 (n=5)
Rutin	363 ± 98 (n=5)

Kv1.1 dose responses for tannic acid, gallic acid, and rutin. Values indicate mean ± SEM.

KV1.2	**EC50 (nM)**
Tannic acid	222 ± 45 (n=5)
Gallic acid	*n.a* (n=5)
Rutin	855 ± 96 (n=5)

Kv1.2 dose responses for tannic acid, gallic acid, and rutin. Values indicate mean ± SEM. *n.a* = not applicable.

Figure 5

	$V_{0.5}$ Normalized tail current (mV)	Slope (mV)	E_M (mV)
Control	-31.61 ± 1.35	5.87 ± 1.19	-43.00 ± 0.97
1:50 Bladderwrack Kelp	-46.71 ± 2.07 (*p*=0.0005; n=5)	3.99 ± 1.63 (*p*=0.3813; n=5)	-54.40 ± 0.84 (<0.0001; n=5)

Statistics versus KV1.1/KV1.1-L155P in absence of Bladderwrack Kelp. Values indicate mean ± SEM.

	$V_{0.5}$ Normalized tail current (mV)	Slope (mV)	E_M (mV)
Control	-35.76 ± 0.92	5.88 ± 0.79	-45.80 ± 0.76
1:50 Pacific Ninebark Root	-40.80 ± 0.98 (*p*=0.0057; n=5)	5.23 ± 0.90 (*p*=0.6023; n=5)	-48.80 ± 1.23 (*p*=0.0046; n=5)

Statistics versus KV1.1/KV1.1-L155P in absence of Pacific Ninebark Root. Values indicate mean ± SEM.

	$V_{0.5}$ Normalized tail current (mV)	Slope (mV)	E_M (mV)
Control	-22.20 ± 0.62	4.76 ± 0.55	-38.40 ± 0.31
1:50 Pacific Ninebark Leaves	-32.30 ± 0.90 (<0.0001; n=5)	6.48 ± 0.78 (*p*=1134; n=5)	-48.00 ± 0.23 (*p*=0.0036; n=5)

Statistics versus KV1.1/KV1.1-L155P in absence of Pacific Ninebark Leaves. Values indicate mean ± SEM.

	$V_{0.5}$ Normalized tail current (mV)	Slope (mV)	E_M (mV)
Control	-36.99 ± 0.41	5.00 ± 0.35	-46.20 ± 0.68
1:50 Nettle	-39.21 ± 0.55 (*p*=0.0133; n=5)	3.88 ± 0.60 (*p*=0.1546; n=5)	-49.00 ± 0.20 (*p*=0.0348; n=5)

Statistics versus KV1.1/KV1.1-L155P in absence of Nettle. Values indicate mean ± SEM.

Figure 6

	$V_{0.5}$ Normalized tail current (mV)	Slope (mV)	E_M (mV)
Control	-11.63 ± 1.11 (n=5)	7.55 ± 2.83 (n=5)	-30.80 ± 0.43
1:50 Bladderwrack Kelp	-29.20 ± 0.65 (<0.0001; n=5)	6.52 ± 0.80 (*p*=0.7415; n=5)	-47.80 ± 0.84 (*p*=0.0025; n=5)

Statistics versus Kv1.1-E283K in absence of bladderwrack kelp. Values indicate mean ± SEM.

	$V_{0.5}$ Normalized tail current (mV)	Slope (mV)	E_M (mV)
Control	-4.98 ± 0.47 (n=5)	4.14 ± 1.59 (n=5)	-22.00 ± 0.28
1:50 Pacific Ninebark Root	-19.24 ± 0.87 (<0.0001; n=5)	3.27 ± 1.63 (*p*=0.7124; n=5)	-34.20 ± 0.85 (*p*=0.0009; n=5)

Statistics versus Kv1.1-E283K in absence of Pacific Ninebark root. Values indicate mean ± SEM.

	$V_{0.5}$ Normalized tail current (mV)	Slope (mV)	E_M (mV)
Control	-7.80 ± 1.03 (n=5)	7.03 ± 3.93 (n=5)	-24.80 ± 1.24
1:50 Pacific Ninebark Leaves	-19.88 ± 0.76 (<0.0001; n=5)	6.05 ± 2.81 (*p*=0.8448; n=5)	-35.00 ± 0.84 (*p*=0.0003; n=5)

Statistics versus Kv1.1-E283K in absence of Pacific Ninebark Leaves. Values indicate mean ± SEM.

	$V_{0.5}$ Normalized tail current (mV)	Slope (mV)	E_M (mV)
Control	-5.84 ± 1.09 (n=6)	6.18 ± 0.95 (n=6)	-35.40 ± 0.74
1:50 Nettle	-24.17 ± 0.55 (<0.0001; n=6)	5.00 ± 0.47 (*p*=0.2268; n=6)	-47.00 ± 0.83 (<0.0001; n=6)

Statistics versus Kv1.1-E283K in absence of Nettles. Values indicate mean ± SEM.

	**Control Tau_{Act} (ms)**	**1 µM Tannic acid Tau_{Act} (ms)**
-20 mV	19.99 ± 7.47	41.32 ± 8.47 (*p*=0.0962; n=5)
-10 mV	10.66 ± 4.72	27.06 ± 7.08 (*p*=0.0955; n=5)
0 mV	8.72 ± 4.64	21.12 ± 4.35 (*p*=0.0872; n=5)
+10 mV	4.26 ± 1.26	17.43 ± 3.97 (*p*=0.0265; n=5)
+20 mV	3.86 ± 1.20	12.72 ± 2.85 (*p*=0.0324; n=5)
+30 mV	3.40 ± 1.06	10.63 ± 2.79 (*p*=0.0587; n=5)
+40 mV	3.09 ± 0.91	10.04 ± 2.65 (*p*=0.0565; n=5)

Statistics versus Kv1.1-E283K in absence of bladderwrack kelp. Values indicate mean ± SEM.

	**Control Tau_{Deact} (ms)**	**1 µM Tannic acid Tau_{Deact} (ms)**
-80 mV	0.88 ± 0.32	1.50 ± 0.57 (*p*=0.3632; n=8)
-70 mV	0.38 ± 0.15	1.38 ± 0.56 (*p*=0.1228; n=8)
-60 mV	0.32 ± 0.09	1.23 ± 0.52 (*p*=0.1259; n=8)
-50 mV	0.20 ± 0.05	1.04 ± 0.44 (*p*=0.0986; n=8)
-40 mV	0.19 ± 0.05	0.88 ± 0.36 (*p*=0.0979; n=8)
-30 mV	0.19 ± 0.05	0.79 ± 0.32 (*p*=0.1044; n=8)
-20 mV	0.19 ± 0.06	0.87 ± 0.36 (*p*=0.1025; n=8)

Statistics versus Kv1.1-E283K in absence of bladderwrack kelp. Values indicate mean ± SEM.

Figure 7

	$V_{0.5}$ Normalized tail current (mV)	Slope (mV)	E_M (mV)
Control	*n.a*	*n.a*	-41.20 ± 1.84
1 µM Gallic acid	*n.a*	*n.a*	-47.00 ± 2.56 (*p*=0.1067; n=5)

Statistics versus Kv1.1/Kv1.1-G311D in absence of Gallic acid. Values indicate mean ± SEM. *n.a* = not applicable.

	$V_{0.5}$ Normalized tail current (mV)	Slope (mV)	E_M (mV)
Control	*n.a*	*n.a*	-25.40 ± 1.37
1 µM Gallic acid	*n.a*	*n.a*	-33.00 ± 1.09 (*p*=0.0131; n=5)

Statistics versus Kv1.1/Kv1.1-L328V in absence of Gallic acid. Values indicate mean ± SEM. *n.a* = not applicable.

	$V_{0.5}$ Normalized tail current (mV)	Slope (mV)	E_M (mV)
Control	*n.a*	*n.a*	-41.40 ± 1.98
1 µM Gallic acid	*n.a*	*n.a*	-46.60 ± 0.60 (*p*=0.0289; n=5)

Statistics versus Kv1.1/Kv1.1-V408A in absence of Gallic acid. Values indicate mean ± SEM. *n.a* = not applicable.

	$V_{0.5}$ Normalized tail current (mV)	Slope (mV)	E_M (mV)
Control	-12.01 ± 0.57	4.52 ± 0.51	-33.80 ± 0.18
1 µM Gallic acid	-27.50 ± 0.51 (<0.0001; n=4)	5.70 ± 1.12 (*p*=0.3772; n=4)	-47.80 ± 0.65 (*p*=0.0005; n=4)

Statistics versus Kv1.1/Kv1.1-E283K in absence of Gallic acid. Values indicate mean ± SEM.

Figure 8

	$V_{0.5}$ Normalized tail current (mV)	Slope (mV)	E_M (mV)
Control	-23.84 ± 0.45	4.30 ± 0.35	-34.89 ± 0.98 (n=9)
1 µM Gallic acid	-30.59 ± 0.60 (<0.0001; n=9)	4.84 ± 0.56 (*p*=0.4278; n=9)	-45.00 ± 0.78 (*p*=0.0002; n=9)
10 µM Gallic acid	-36.38 ± 0.62 (<0.0001; n=9)	4.76 ± 0.51 (*p*=0.4692; n=9)	-48.89 ± 0.66 (<0.0001; n=9)
100 µM Gallic acid	-37.93 ± 0.62 (<0.0001; n=9)	4.68 ± 0.55 (*p*=0.5695; n=9)	-50.11 ± 0.69 (<0.0001; n=9)

Statistics versus Kv1.1/Kv1.1-L155P in absence of Gallic acid. Values indicate mean ± SEM.

Figure 9

	$V_{0.5}$ Normalized tail current (mV)	Slope (mV)	E_M (mV)	Current-fold change -30 mV
Control	-11.21 ± 0.61	5.13 ± 0.60	-27.00 ± 0.35	*n.a*
1 µM Tannic acid	-22.75 ± 1.34 (<0.0001; n=6)	8.70 ± 1.19 (*p*=0.0301; n=6)	-44.83 ± 0.14 (<0.0001; n=6)	4.69 ± 1.69 (<0.0001; n=6)

Statistics versus Kv1.1-E283K in absence of Tannic acid. Values indicate mean ± SEM. *n.a* = not applicable.

	Control Tau_{Act} (ms)	1 µM Tannic acid Tau_{Act} (ms)
-20 mV	24.46 ± 3.97	31.31 ± 2.98 (*p*=0.2679; n=6)
-10 mV	15.41 ± 2.70	20.71 ± 2.35 (*p*=0.1701; n=6)
0 mV	11.02 ± 1.62	15.70 ± 1.40 (*p*=0.0543; n=6)
+10 mV	8.90 ± 0.89	12.94 ± 1.05 (*p*=0.0153; n=6)
+20 mV	7.43 ± 0.68	11.30 ± 0.89 (*p*=0.0068; n=6)
+30 mV	6.35 ± 0.66	10.16 ± 0.83 (*p*=0.0053; n=6)
+40 mV	5.50 ± 0.69	9.46 ± 0.83 (*p*=0.0046; n=6)

Statistics versus Kv1.1-E283K in absence of Tannic acid. Values indicate mean ± SEM.

	**Peak current at -40 mV (µA)**
Control	0.02 ± 0.01
0.1 µM	0.02 ± 0.01 (>0.9999; n=5)
1 µM	0.02 ± 0.01 (>0.9999; n=5)
10 µM	0.02 ± 0.01 (>0.9999; n=5)
30 µM	0.02 ± 0.01 (>0.9999; n=5)
100 µM	0.03 ± 0.01 (*p*=0.496; n=5)

Statistics versus Kv1.1-E283K in absence of Tannic acid. Values indicate mean ± SEM.

Figure 10

	**EC50 (nM)**
Kv1.1	379 ± 28 (n=5)
Kv1.1-3M	*n.a*

Kv1.1 vs Kv1.1-3M dose responses for gallic acid. Values indicate mean ± SEM. *n.a* = not applicable.

	**EC50 (nM)**
Kv1.1	18 ± 6 (n=5)
Kv1.1-3M	345 ± 38 (n=5)

Kv1.1 vs Kv1.1-3M resting membrane potential (E_M) dose responses for gallic acid. Values indicate mean ± SEM.
