# Supplementary material for: Native American ataxia medicines rescue ataxia-linked mutant potassium channel activity via binding to the voltage sensing domain
Source: Nat Commun. 2023 Jun 6;14:3281. doi: 10.1038/s41467-023-38834-6 (PMC10244465; doi:10.1038/s41467-023-38834-6)
Supplement: Supplementary file 3 — Description of Additional Supplementary Files [file 41467_2023_38834_MOESM3_ESM.pdf]

Supplementary Movie 1: Gallic acid binding to the wild-type Kv1.1 VSD. Configurations from MD simulation 300-ns trajectories taken every 1 ns. The wild-type Kv1.1 voltage sensing domain (VSD) is shown in ribbon representation and the gallic acid molecule and VSD residues in its immediate neighborhood are shown in licorice representation colored by atom (C, silver; N, blue; O, red; H, white).

Supplementary Movie 2: Gallic acid interaction with the Kv1.1-3M VSD. Configurations from MD simulation 300-ns trajectories taken every 1 ns. The Kv1.1-3M VSD is shown in ribbon representation and the gallic acid molecule and VSD residues in its immediate neighborhood are shown in licorice representation colored by atom (C, silver; N, blue; O, red; H, white).
